# Is there a role for voltage-gated Na^+^ channels in the aggressiveness of breast cancer?

**DOI:** 10.1590/1414-431X20176011

**Published:** 2017-06-05

**Authors:** P. Rhana, R.R. Trivelato, P.S.L. Beirão, J.S. Cruz, A.L.P. Rodrigues

**Affiliations:** 1 Laboratório de Câncer de Mama, Canais Iônicos e AMP Cíclico, Faculdade de Ciências Humanas, Sociais e da Saúde, Universidade FUMEC, Belo Horizonte, MG, Brasil; 2 Laboratório de Membranas Excitáveis e de Biologia Cardiovascular, Departamento de Bioquímica e Imunologia, Instituto de Ciências Biológicas, Universidade Federal de Minas Gerais, Belo Horizonte, MG, Brasil

**Keywords:** Breast cancer, Ion channels, Metastasis, Nav1.5 channel

## Abstract

Breast cancer is the most common cancer among women and its metastatic potential is responsible for numerous deaths. Thus, the need to find new targets for improving treatment, and even finding the cure, becomes increasingly greater. Ion channels are known to participate in several physiological functions, such as muscle contraction, cell volume regulation, immune response and cell proliferation. In breast cancer, different types of ion channels have been associated with tumorigenesis. Recently, voltage-gated Na^+^ channels (VGSC) have been implicated in the processes that lead to increased tumor aggressiveness. To explain this relationship, different theories, associated with pH changes, gene expression and intracellular Ca^2+^, have been proposed in an attempt to better understand the role of these ion channels in breast cancer. However, these theories are having difficulty being accepted because most of the findings are contrary to the present scientific knowledge. Several studies have shown that VGSC are related to different types of cancer, making them a promising pharmacological target against this debilitating disease. Molecular biology and cell electrophysiology have been used to look for new forms of treatment aiming to reduce aggressiveness and the disease progress.

## Introduction

**Figure 1.**
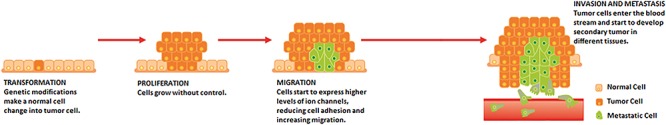
Tumorigenesis process. The interaction between DNA and different carcinogenic factors can cause mutations in genes that are responsible for controlling several important cellular functions, such as the regulation of apoptosis. The injured cell then has a lack of proliferation control, which leads to the formation of tumors. The tumor can present a higher degree of aggressiveness, in which tumor cells have characteristics like the loss of cell adhesion and increase in expression levels of ion channels. In some aggressive cancers, cells can reach the blood stream and start the development of a cancer in new sites throughout the body (metastasis). (Figure adapted with permission from Araújo P et al. *Ciência Hoje* 2014; 54: 36-39. (78)).

Cancer is one of the main causes of death worldwide, and understanding the origins and progression of the disease is essential for better treatments and diagnosis. Death is mainly caused by metastasis, in a stage where tumor cells disassociate from each other, destroy the basal membrane, and enter the blood stream, forming secondary tumors in other parts of the body (Figure 1) (1–4).

Tumors are classified into benign and malignant. A benign tumor is characterized by a localized mass of cells, which multiplies slowly and resembles the original tissue, and normally is not life threatening. A malignant tumor shows a higher degree of mutations and autonomy, can invade adjacent tissue and present migration of tumor cells to new places in the body, forming metastatic tumors. Metastasis is caused by the loss of cell adhesion, which leads to their migration to the extracellular matrix where they can reach the blood or lymphatic vessels. The rate of metastasis increases with the degree of aggressiveness of the tumor cells. This process is affected by different molecular and cellular factors, and also by the microenvironment in which the tumor is located. All these factors will determine the occurrence or absence of metastasis (2,3).

Ion channels, which are transmembrane proteins that function as gates that control the flux of ions across the cell membrane, have been associated with tumorigenesis and tumor progression, although their role in those processes is not totally understood. Many studies have demonstrated the participation of voltage-gated Na^+^ channels (VGSC) in the progression of different tumors, such as prostate cancer, small cell lung cancer, breast cancer and others, linking VGSC to the invasion capacity of tumor cells ([Bibr B05] 4–9). With regard to breast cancer, it has been found that VGSC are more expressed in cells with a high metastatic capacity, such as MDA-MB-231 cell line, when compared to weakly metastatic breast cancer cells, as MCF-7 cell line (10–13). It has been shown that the invasion capacity of MDA-MB-231 was reduced by approximately 30% when Na^+^ currents were blocked with tetrodotoxin (TTX), a potent VGSC blocker (14). Results reported by Fraser and others (15) show that Na^+^ channels’ activity was associated with the increase in cellular motility, endocytosis and invasion of metastatic human breast cancer cells. They also revealed that expression of a “neonatal” splicing form of VGSC was increased in these cells and was directly associated with the metastatic potential.

Taking into account the role of VGSC in the progression of breast cancer, this protein should be considered as a new target for developing anti-cancer therapies. This paper reviews recent information about these ion channels and their participation in the tumorigenesis process. We also discuss the possibilities of VGSC being a potential therapeutic target.

## Cancer statistics

Breast cancer is the second most common cancer in the world and the most frequent among women. Breast cancer is the fifth most common cause of overall death from cancer, only behind lung, liver, colorectal and stomach. Predictive statistics indicate that the number of new cases will rise from 14 million in 2012 to 22 million in the next two decades (16,17), and the number of deaths will be increased by 45% until 2030 (18).

It is believed that the first event that occurs in the cells during carcinogenesis is the accumulation of DNA genetic alterations, which results in erroneous regulation of expression levels and/or patterns of certain proteins. Among these alterations are mutations in proto-oncogenes and tumor suppressor genes, and hyper- or hypo-methylation of DNA, which are caused by interactions with chemical, biological and physical carcinogenic factors, such as radiation, tobacco, alcohol, and infections by some viruses and bacteria (1,17,19,20).

## Voltage-gated Na^+^ channels

Ion channels are signaling molecules expressed throughout the human body, being responsible for processes such as cell proliferation, solute transport, maintenance of membrane potential, nerve signaling and control of muscle contraction, secretion, invasion and many other activities (15,21–24). Consequently, alterations in the expression and/or function of these proteins may lead to the development of different diseases, like cardiac arrhythmias, epilepsy, multiple sclerosis and the progression of different types of cancer to advanced stages (3,15).

Among the existing ion channels, VGSC are mostly expressed in excitable cells, including neurons and muscle cells, and are responsible for initiating and propagating electrical signals. Studies have shown that these channels are also present in non-excitable cells, although their physiological function in these cells is not yet well understood (8,11,24,25).

**Figure 2.**
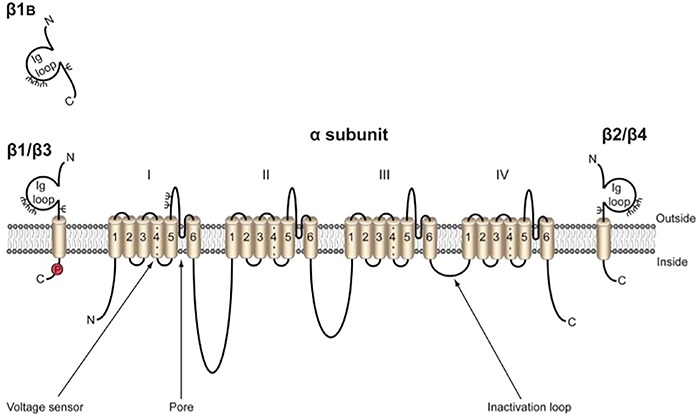
Voltage-gated Na^+^ channel. The pore-forming α subunit is composed by four homologous domains with 6 transmembrane segments (S1-S6). The voltage sensor is located in S4. The β subunits present an immunoglobulin loop on the extracellular domain, a transmembrane domain and a C-terminal intracellular domain. β1 and β3 connect non-covalently with α subunit, whereas β2 and β4 connect through disulfide bonds. (Figure published with permission from Brackenbury WJ and Isom LL. *Front Pharmacol* 2011; 2: 53, doi: 10.3389/fphar.2011.00053. (65)).

The VGSC are composed of a pore-forming α subunit that can be associated with one or more β subunits (Figure 2). Na^+^ channel α subunits are composed of four homologous domains, each of which has six transmembrane segments. The VGSC family has 9 members, Nav1.1 through Nav1.9, encoded by the genes *SCN1A-SCN5A* and *SCN8A-SCN11A*. As for the β subunits, five members have been found, β1 to β4 and β1B (an alternative splicing of β1), and are encoded by the genes *SCN1B* through *SCN4B.* These are mainly composed of an extracellular N-terminal segment, a single transmembrane segment and a short intracellular segment. Pore-forming subunits may be expressed alone, since their operation is independent of the presence of the β subunits. However, these subunits are essential, as they are responsible for modulating the function of the channel (opening, closing and inactivating it) and allow the association with cell adhesion molecules, extracellular matrix and cytoskeleton (11,26,27).

### VGSC and intracellular acidification

**Figure 3.**
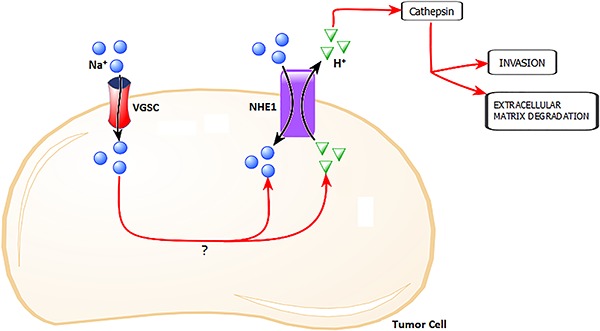
pH regulation affects tumor invasion process. The opening of voltage-gated Na^+^ channels (VGSC) causes an increase of the intracellular Na^+^ concentration and the activation of Na^+^/H^+^-1 exchanger (NHE1). The efflux of protons lead to a high extracellular pH and a low intracellular pH, the latter being a favorable environment for the activation of cathepsins (enzymes that act in the extracellular matrix degradation). This process enhances the invasion capacity of tumor cells.

In recent years, many studies with cell cultures and analysis of biopsy tissues have provided evidence that VGSC are responsible for increasing the invasive potential of tumor cells, participating in the processes of galvanotaxis, cellular motility, migration, and others. Inhibition of VGSC has been linked with reduction of metastatic behavior (4–8,28). The main question is how the influx of Na^+^ through these channels increases the invasive and metastatic potential of tumors. Different groups have presented distinct theories (associated with pH, gene expression and intracellular Ca^2+^) in an attempt to better understand the role of these channels and allow the development of new antineoplastic drugs (9,27). Influx of Na^+^ through the Nav1.5 channels resulted in intracellular alkalinization and consequent acidification of the extracellular space close to the cell membrane (6,29,30). The mechanism that explains pH variation is associated with co-expression of Na^+^/H^+^ (NHE)-1 exchanger, an important protein that transports hydrogen ions. The influx of Na^+^ through VGSC increases the efflux of H^+^, resulting in a high intracellular and a low extracellular pH. Low pH favors the activation of cathepsins B and S, which are proteolytic enzymes responsible for the extracellular matrix degradation (31,32), thus enhancing pH-dependent extracellular matrix degradation and invasion (Figure 3). In another study, Brisson et al. (33) demonstrated that expression of Nav1.5 also promotes modification of the F-actin network and enhances NHE-1 activity in breast cancer cells, resulting in increased invasiveness of malignant cells. This proposition of how VGSC and NHE-1 affect extracellular pH is opposite to the traditional view of how these two proteins interact. It is important to note, however, that cancer cells might have a completely different organization and functioning than normal cells.

Another important point that is not taken into account is the relative contribution of the endo/lysosome H^+^ extrusion to the formation of an acidic tumor environment and to increase cathepsins activity. Endosomes are spherical structures formed from the cellular membrane, which contain approximately 40 hydrolytic enzymes capable of digest cellular components, like mitochondria, intracellular vesicles and even the whole cell. After its constitution, the endosome can be transformed in lysosome or recycled back to cell membrane (34). Lysosome formation is one of the main roles of endosomes. According to Carrithers et al. (35) and Black et al. (36), the lysosomal system is complex and highly dynamic. It begins as an early endosome and through a maturation process turns into a late endosome and then to a lysosome. Different changes occur in this process, such as pH reduction, reception of vesicles coming from the Golgi apparatus, and the activation of lysosomal enzymes (35,37).

The acidic environment of endolysosomes is attributed to V-ATPase, proteins capable of using the energy of ATP hydrolysis to transport H^+^ through intracellular membrane compartments. Recently, it was demonstrated that Nav1.5, classically a cardiac isoform of VGSC, is also present in late endosomes being responsible for an extra-acidification of this vesicle. The authors suggested a model to explain how these ion channels work and the general idea is that they provide a passage for positive charges (Na^+^) from inside of the endosome to cytoplasm. This transport enables the entry of more protons into the endosome through voltage-dependent channels CIC or H^+^-ATPase pump (35).

### Lysosomal trafficking and cathepsins activation

It is well known that lysosome trafficking is altered in tumor cells (38). Therefore, the actual contribution of this process to the acidification of extracellular environment and, consequently, the cathepsins role during cancer progression must be considered and further investigated.

Cathepsins are lysosomal peptidases that participate in the intracellular protein catabolism. These enzymes are synthesized as inactive zymogens and are activated after the break of a pro-peptide by another protease or due to low pH, the optimal environment for cathepsins action. Protein degradation is involved in different cellular processes, physiological or pathological, such as autophagy, antigen presentation, cellular stress signaling and apoptosis. Besides being commonly associated with tumor progression because of their role in increasing extracellular matrix degradation, cathepsins are also involved in apoptosis regulation (38).

Apoptosis induction by cathepsins can be through the extrinsic or death receptor pathway or through the intrinsic or mitochondrial pathway. The first pathway is activated by specific ligands of death receptors and posterior activation of caspase 8, which will cleave Bid, a pro-apoptotic molecule. The cleavage of Bid will generate a truncated form of this molecule (tBid), capable of inducing mitochondrial outer membrane permeabilization and, consequently, the release of cytochrome c. Both pathways are connected through Bid, but for the intrinsic pathway the stimuli will be the presence of reactive oxygen species, which are produced during cellular stress and may cause lysosomal membrane permeabilization, a non-proteolytic event that releases cathepsins into intracellular milieu. Cathepsins will not only stimulate the cleavage of Bid but also the inactivation of anti-apoptotic proteins, such as Bcl-2 (39,40).

### Control of intracellular pH

The concentration of intracellular H^+^ has an important role in different cellular processes, because protein structure and function depend on optimal pH. Cellular compartmentalization is necessary to keep environmental conditions for individual pathways and prevent cellular processes that would cause functional changes (41). Cells have the tendency to acidify due to products of metabolic reactions and to electrical potential across the membrane, which pulls positive ions into the intracellular space. Therefore, the removal of protons and their equivalents is a constant process (42).

Many transporters, which are expressed on cellular membrane and organelles of secretory and endocytic pathways, stringently control intracellular pH. Among them are V-ATPase (as mentioned above), NHE, Na^+^-coupled HCO3- transporters (NBC) and ion channels (42,43). Ion channels are functionally present on membranes of the aforementioned organelles and also are involved with the ionic homeostasis. There are common challenges in studying channels from different intracellular organelles. Unlike plasma membrane channels that have been unambiguously defined, the basic information for most organelles has yet to be established, including luminal ionic composition, organelle membrane potential, and lipid composition of the organelle membrane. Although the importance of lysosomal ionic flux has long been appreciated, the ion channels responsible for lysosomal Na^+^, K^+^, Ca^2+^, Cl^-^ and H^+^ fluxes are only beginning to be discovered (44) and more studies are clearly necessary to explain this important issue.

### Gene network and ion channels

Another hypothesis that tries to explain the role of the ion channels in the physiopathology of cancer is based on how VGSC can regulate the expression of certain genes, called invasion gene network (3,27). It is well known that Na^+^ channels are capable of regulating gene expression in excitable cells and cancer cells (23,45–47). Furthermore, *SCN5A* gene is a key regulator of this invasion gene network, suggesting that Nav1.5 may function as early entry points of invasion signaling mechanisms. At least in colon cancer, Nav1.5 may regulate invasion by this mechanism in addition to extracellular acidification (48). The challenge is to understand how the activity of ion channels can regulate transcription in cancer cells.

### Ca^2+^ and Na^+^ crosstalk

**Figure 4.**
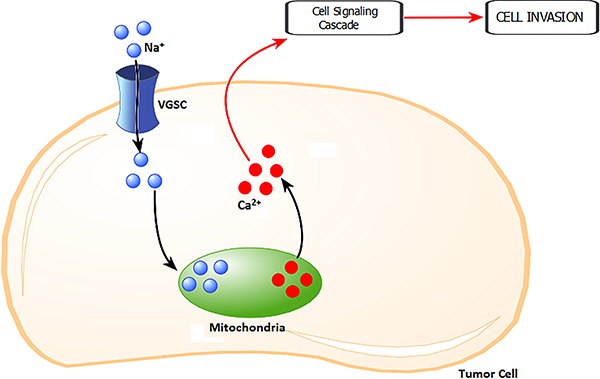
Regulation of intracellular Ca^2+^ through voltage-gated Na^+^ channels (VGSC). After the activation of VGSC, a quick absorption of Na^+^ ions and release of Ca^2+^ ions by the mitochondria occur. Somehow, these events activate a cell-signaling cascade to increase the formation of podosomes, consequently, increasing the cell invasion ability of the tumor.

A third possibility is related to the regulation of intracellular Ca^2+^ through VGSC. In excitable and non-excitable cells, influx of Na^+^ may result in an increase of intracellular Ca^2+^ levels through the activation of voltage-gated Ca^2+^ channels (49). In addition to the plasma membrane, voltage-gated Ca^2+^ channels are present in internal membranes such as podosomes and endosomes of cancer cells and macrophages (13,50,51). In THP-1 macrophages and HTB-66 melanoma cells, Nav1.6 channel is expressed in vesicular structures near podosomes regions. Surprisingly, the activation of these cells mediated by VGSC show a rapid uptake of Na^+^ by mitochondria and subsequent release of Ca^2+^ into the cytosol. It is proposed that this process increases the formation of podosomes/invadopodia, leading to increased cell invasion (Figure 4) (50). However, it is not clear how VGSC present in vesicular membranes are gated and/or whether they interact with VGSC present in the plasma membrane (9). In vascular endothelial cells the elevation of intracellular Ca^2+^ requires Na^+^ influx and in turn activates PKC and extracellular signal-regulated kinase (ERK)1/2, potentiating angiogenic functions including proliferation, differentiation and adhesion (52).

### Back to VGSC

In cancer cells, there is no specific pattern for subunits, since different α subunits are expressed in different types of cancer. For breast cancer, the subtype most commonly found so far is the Nav1.5, which is encoded by the gene *SCN5A* and is found in two different forms: 1) the neonatal (nNav1.5), and 2) the adult splicing variant (9–11). An alternative splicing can occur between two different exons present in exon 6 of *SCN5A* (5' exon and 3' exon) and the difference will be the presence or absence of an aspartate residue at position 7 in the exon 6 (3). Through studies in rat brain, it was possible to conclude that the transcription containing the 5' exon was more common at birth but was quickly replaced by the 3' exon. This splicing pattern has been found in studies with Nav1.1, Nav1.2, Nav1.3, Nav1.5, Nav1.6 and Nav1.7 (53–58).

In a study to determine which Na^+^ channels have functional expression in different types of cancer cell lines and cancer primary cultures, the neonatal Nav1.5 channel isoform was found in breast cancer cells with high metastatic potential. This protein was identified in biopsy samples and its increased expression could be associated with patients with lymph node metastasis. This indicates that the neonatal isoform is significantly up-regulated in breast cancer cells and potentiates cell processes involved in metastasis (15).

Another observation was that the majority of expressed functional channels were resistant to TTX, a specific Na^+^ channel blocker (15). Experiments performed with breast, prostate and lung cancers using TTX resulted in the suppression of a variety of behaviors specific to highly metastatic cells, such as invasion (14,59,60), lateral motility (61), adhesion (62), migration (45), galvanotaxis (63) and endocytosis (64). However, the importance and function of β subunits in increased cell aggressiveness remains to be explored (23).

The expression of β subunits and their participation in cell migration and adhesion in two cell types, MDA-MB-231 and MCF-7, demonstrated that MCF-7 cells had higher expression levels of proteins encoded by *SCN1B, SCN2B* and *SCN4B* genes compared to MDA-MB-231 cells. However, for both cell lines the most common subunit was β1. Silencing β1 expression resulted in decreased cell adhesion and higher migration in 3D cultures for MCF-7 cells (23).

The same study showed that overexpression of β1 subunit in MDA-MB-231 cells increased Na^+^ current, the length of cell processes and the intercellular adhesion, in addition to reducing the lateral motility and proliferation. Considering all these findings, the authors were able to conclude that the expression of β1 enhances cell adhesion and reduces the migration of cells in breast cancer. It is important to note that the effects on the adhesive capacity of these cells can occur independently of changes in membrane excitability, confirming that β subunits have the ability to operate in the absence of the α subunit (23).

**Figure 5.**
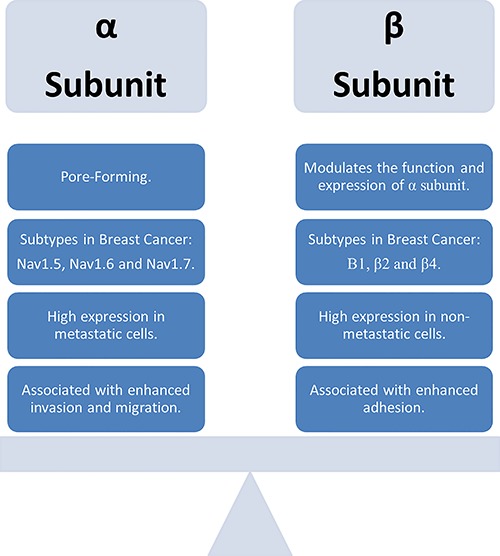
Overview of the main differences of α and β subunits of the voltage-gated Na^+^ channels in tumor cells.

In summary, the β subunits appear to have a role in the regulation of several cellular processes including migration, adhesion, cell proliferation and resistance to apoptosis (65–68). Moreover, these functions appear to have opposite effects to the regulation carried on by α subunits (23). As we have seen previously, α subunits have greater expression in highly metastatic cells and can increase the ability of invasion and migration. β subunits are expressed in cells with little metastatic capacity and are able to modulate Na^+^ influx, increase cell adhesion and the extension processes, and also regulate different activities such as migration and α subunit mRNA expression (Figure 5) (11,65).

## Therapeutic approaches

Each cancer should be considered individually when deciding on the most appropriate treatment procedure. Usually, a combination of treatments is used aiming at the cure or to prolong survival while improving the patient's quality of life. The most commonly used treatments are surgical removal of the tumor, chemotherapy and radiotherapy. Surgery is the most effective treatment when the total removal of the tumor is possible, but in most cases, it is combined with chemotherapy and/or radiotherapy (17).

Chemotherapy is the utilization of various drugs with the objective of killing the cancer cells, although it also causes side effects on normal cells. The mechanism of action of chemotherapic drugs is by the interference in the cell cycle and in cell proliferation. Metabolic differences and faster proliferation rate make cancer cells more susceptible to the drugs. However, normal cells with fast rates of renewal are also affected and generate side effects as hair loss, anemia, immunologic depression, nausea, vomiting, dizziness, weakness and others (69). The dose needed to achieve a balance between the maximal toxic effect to malignant cells with the minimal effect on normal cells is the challenge in chemotherapy.

Radiotherapy employs the emission of ionizing radiation that releases free electrons in affected tissues and causes alterations in the DNA, triggering different cell signaling and causing tumor destruction. The effectiveness of this treatment depends on various factors such as tumor sensitivity to radiation, tumor location and the amount of radiation. The adverse effects of this treatment also result from the injury to normal cells, but since the treatment is very localized it is usually well tolerated by patients when the principles of total dose per treatment and fractionated application are respected (69).

The important role that VGSC seems to have in metastatic cells and unsatisfactory clinical results of regular treatments, make these ion channels a real possibility for the development of new methods of diagnosis and perhaps a more effective therapeutic approach. Among the pharmacological tools presently available to target these molecules are drugs that block the functioning of ion channels. One main blocker that has been studied is TTX, a well-known Na^+^ channel blocker (9).

Each α subunit isoform has a particular sensitivity to TTX, in addition to other pharmacological and physiological properties. The Nav1.1, Nav1.4, Nav1.6 and Nav1.7 Na^+^ channels are part of a group that exhibits the greatest sensitivity to this toxin, since a nanomolar (nM) concentration is necessary to block these channels. As for the Nav1.5, Nav1.8 and Nav1.9 Na^+^ channels, the necessary concentrations of TTX to achieve full blocking effect are in the micromolar (µM) range (11,25). However, the toxicity of this toxin to the human body and the low sensitivity of some of its isoforms impede TTX to be used systemically as an anti-metastatic treatment (25). Ranolazine and phenytoin, examples of other Na^+^ channel blockers, are now being tested for use in cancer treatment. Thus far, the results with these drugs show an inhibition of metastatic behavior in *in vitro* experiments, reducing invasion and metastasis (13,28,70–72).

Another form of therapy is genetic silencing, which uses a technique where double-stranded RNAs (siRNA) are synthesized artificially. The ideal target of siRNAs are genes that are expressed or abnormally regulated only in the tumor cells, or genes specifically involved in cell proliferation, angiogenesis and/or metastasis (73).

This approach is still being tested for different diseases with variable results (74,75). The U.S. Food and Drug Administration approved a new antineoplastic siRNA for clinical phase I studies against solid tumors (76). In breast cancer, the *in vitro* study for gene silencing of nNav1.5 showed very good results. The interruption of the function and expression of these channels in MDA-MB-231 breast cancer cells caused a significant suppression in the migratory capacity (about 50%) of these cells (77). However, the reproduction of these results *in vivo* is much more complicated and the clinical use of this new treatment depends on the stability of these molecules and the non-suppression of other targets (3).

## Conclusion and future perspectives

Studies on cancer and ion channels started in the 1990s with prostate cancer, followed by a series of investigations focusing on the pathophysiological role of ion channel expression in different types of cancer. Afterwards, more studies were performed with different cell types, and upregulation of voltage-gated Na^+^ channels have been described in cells presenting high metastatic potential. Over the years, new molecular biology techniques have been used to investigate ion channels, revealing the presence of different splice variants and giving a better understanding of the underlying mechanisms responsible for the different behaviors of metastatic cells.

The association between breast cancer and Na^+^ channels, especially voltage-gated channels, was shown to have great importance for cellular events that increase tumors aggressiveness, mainly proliferation, migration, loss of adhesion and galvanotaxis. Some of the proposed theories on how the VGSC relate to the increased aggressiveness of tumors refute all the current knowledge on Na^+^ homeostasis and raise the questions: do cancer cells have a completely different organization compared to normal cells? Can we identifying the mechanisms of a tumor cell looking at the normal cell or should we accept that we are facing the unknown, where cellular structures might have different functions and relations in cancer cells?

Besides being distressful, these differences can also encourage the search of a better understanding for cancer pathophysiology. Further research should be conducted to unravel mechanisms involving VGSC, NHE-1 and pH. Also of importance is determining if the properties associated with β subunits are or not dependent on the α subunits expression.

At this point, no ligand is available that can be used selectively for Nav1.5 channels, which is one of the main targets for new anti-cancer therapeutic approaches. However, new toxins are discovered every day, increasing the existing arsenal that would lead to new and more effective drugs.
